# Graphical Abstract: ChemistryOpen 4/2015

**DOI:** 10.1002/open.201580411

**Published:** 2015-08-21

ChemistryOpen is a multidisciplinary, gold-road, open-access, international forum for the publication of Reviews, Full Papers and Communications from all areas of chemistry and related fields. *ChemistryOpen* also publishes the Thesis Treasury containing summaries of Ph.D. theses and links to the full version via our homepage. Based in Europe, *ChemistryOpen* attracts authors and readers from around the world, as open-access publishing becomes more important in all areas of chemistry. *ChemistryOpen* is coowned by ChemPubSoc Europe and published by Wiley-VCH. Authors can submit their review articles, primary research articles and thesis summaries via our homepage by clicking “submit an article”. All contributions considered suitable for publication are subject to peer review, and if accepted, electronically processed and published online ensuring high quality and short publication times.

## COVER PICTURE

**Figure:**
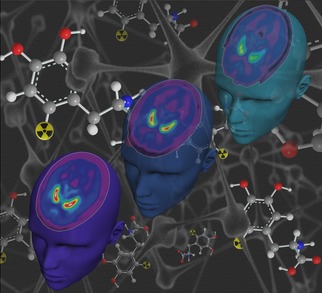


The cover picture shows the differences in brain metabolism of a healthy young and a healthy old subject, as well as a patient suffering from Parkinson's disease (left to right) uncovered by 6-[^18^F]FDOPA-positron emission tomography (PET). Morbus Parkinson occurs when nerve cells that produce dopamine begin to die. The shortage of dopamine leads to movement problems in affected individuals. 6-[^18^F]FDOPA is extensively used to evaluate the progression of Parkinson's disease. Bold stick projections of this PET tracer, as well as a neuronal network, are seen in the background. Unfortunately, conventional procedures to produce 6-[^18^F]FDOPA are cumbersome. Thus, several recent developments aim at the simplification of this radiosynthesis. In our work, we studied the applicability of the recently reported Ni-mediated radiofluorination approach for daily routine production of 6-[^18^F]FDOPA. For more details, see the Full Paper on p. 457 ff.

## COVER PROFILE

B. D. Zlatopolskiy, J. Zischler, E. A. Urusova, H. Endepols, E. Kordys, H. Frauendorf, F. M. Mottaghy, B. Neumaier*

**Figure:**
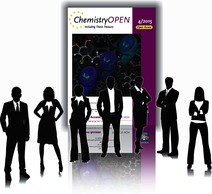


395

A Practical One-Pot Synthesis of Positron Emission Tomography (PET) Tracers via Nickel-Mediated Radiofluorination

“This should facilitate the accessibility of known PET tracers and accelerate the development of novel molecular probes for clinical applications. The ultimate goal of our research is to improve the efficacy of clinical diagnostics and, finally, patient care.“

Learn more about the story behind the research featured on the front cover in this issue's Cover Profile. Read the corresponding article on p. 457 ff.

## NEWS

Spotlights on our sister journals 404–407

## REVIEWS

Y. Zhu,* N. S. Hosmane

**Figure:**
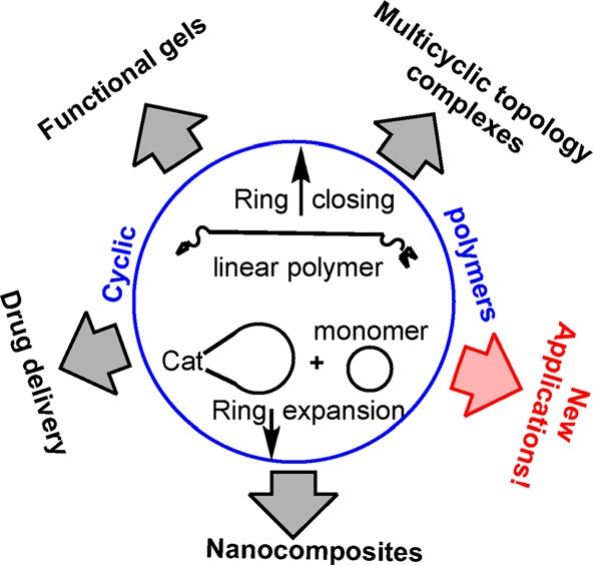


408–417

Advanced Developments in Cyclic Polymers: Synthesis, Applications, and Perspectives

**Round and round they go!** Cyclic polymers have interesting topologies that allow them to have unique physical and biological properties, compared with their linear counterparts. Some examples are cyclic DNA/duplexes, which have therapeutic potential due to their structural and thermal stability. This review summarizes the most common synthetic methods as well as the most interesting properties and applications of these functional materials.

F. Sancenûn,* L. Pascual, M. Oroval, E. Aznar, R. Mart.nez-M.Çez*

**Figure:**
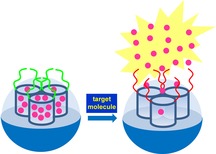


418–437

Gated Silica Mesoporous Materials in Sensing Applications

**Heightened senses!** Capped silica mesoporous supports (SMSs) have found wide application as delivery vehicles due to their ability to release their cargo in response to chemical, biochemical or physical stimuli. Here, a review of the application of gated SMSs in recognition protocols for anions, cations, small molecules, and large biomolecules is presented.

## COMMUNICATIONS

Y. Sun, B. Yoo*

**Figure:**
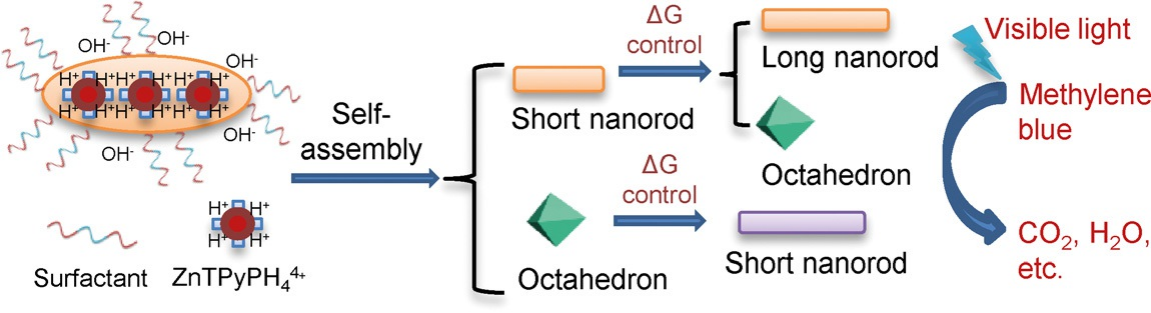


438–442

Morphological Transformation Reactions of Photocatalytic Metalloporphyrin-Containing Coordination Polymer Particles from Seed Structures

**Synthesized from seed structures:** Coordination polymer particles (CPPs) have interesting properties and applications in catalysis, optics, sensing, electronics, photochemistry, and biology. Metalloporphyrin-containing CPPs with nanorod or octahedral structures were synthesized from seed structures through morphological transformation reactions and were used to photocatalyze methylene blue degradation under visible light.

D. Dambournet,* K. W. Chapman, M. Duttine, O. Borkiewicz, P. J. Chupas, H. Groult

**Figure:**
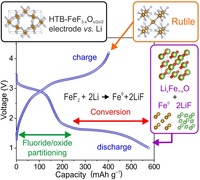


443–447

Lithium Insertion Mechanism in Iron- Based Oxyfluorides with Anionic Vacancies Probed by PDF Analysis

**A battery of tests!** The insertion of lithium into iron-based oxyfluorides yields a composite electrode made of fluoride and oxide phases, as identified by the pair distribution function method. The resulting material shows enhanced intercalation properties relative to the pristine oxyfluoride phase.

Y. Tsuda, A. Shigenaga, K. Tsuji, M. Denda, K. Sato, K. Kitakaze, T. Nakamura, T. Inokuma, K. Itoh, A. Otaka*

**Figure:**
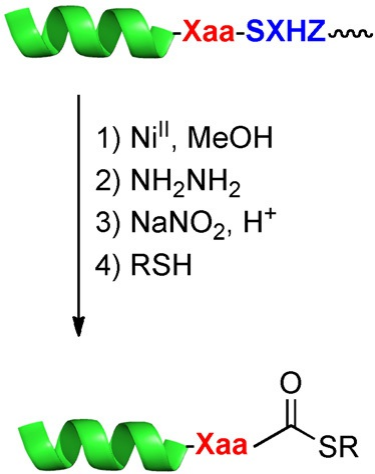


448–452

Development of a Chemical Methodology for the Preparation of Peptide Thioesters Applicable to Naturally Occurring Peptides Using a Sequential Quadruple Acyl Transfer System

**SQATs:** Peptide thioesters are an important compound class in peptide chemistry, but chemistry-based protocols to synthesize natural-peptide-based thioesters are challenging. Here, a sequential quadruple acyl transfer (SQAT) protocol is described for the preparation of thioesters. Importantly, the SQAT system can be applied to naturally occurring peptides, converting them to their corresponding thioesters without the need for an artificial chemical unit.

B. Alameddine, A. H. Rice, C. Luscombe, T. A. Jenny*

**Figure:**
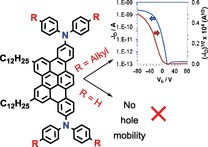


453–456

Synthesis of Arylamine Tribenzopentaphenes and Investigation of their Hole Mobility

**Hole-y compound!** We report the synthesis and hole mobility investigation of two tribenzo[fj,ij,rst]pentaphene (TBP) derivatives bearing two diarylamines at the opposite ends of the aromatic core. The versatile synthesis allows for the decoration of the TBP core with various charge-carrier peripheral groups and alkyl chains, leading to a better hole transport mobility.

## FULL PAPERS

B. D. Zlatopolskiy, J. Zischler, E. A. Urusova, H. Endepols, E. Kordys, H. Frauendorf, F. M. Mottaghy, B. Neumaier*

**Figure:**
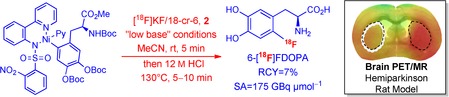


457–462

A Practical One-Pot Synthesis of Positron Emission Tomography (PET) Tracers via Nickel-Mediated Radiofluorination

**Fast and simple:** 6-[^18^F]FDOPA, 6- [^18^F]FMT, and 6-[^18^F]FDA were efficiently prepared via a one-pot two-step procedure using nickel-mediated radiofluorination under “low base” conditions. In a rat model of Parkinson's disease, the biodistribution and, consequently, the imaging property of 6-[^18^F]FDOPA were found to be independent of specific activity.

O. Fu, A. V. Pukin, H. C. Quarles van Ufford, J. Kemmink, N. J. de Mol, R. J. Pieters*

**Figure:**
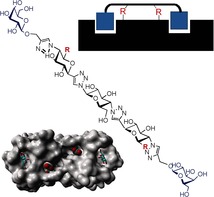


463–470

Functionalization of a Rigid Divalent Ligand for LecA, a Bacterial Adhesion Lectin

**Linkers for lectin ligands!** The *P. aeruginosa* lectin and virulence factor LecA can be efficiently blocked by divalent galactoside ligands containing a rigid spacer. Further functionalization of the spacer was explored in order to foster spacer–protein interactions. Positively and negatively charged groups as well as lipophilic groups were used for this purpose.

O. Fu, A. V. Pukin, H. C. Q. van Ufford, T. R. Branson, D. M. E. Thies-Weesie, W. B. Turnbull, G. M. Visser, R. J. Pieters*

**Figure:**
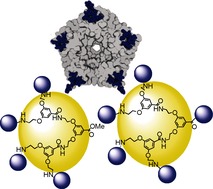


471–477

Tetra- versus Pentavalent Inhibitors of Cholera Toxin

**Carbohydrates combating cholera!** Simultaneous binding of the five B-subunits of the cholera toxin (CTB5) enhances its affinity and facilitates its cellular entry. Thus, blocking the toxin's initial attachment to the cell surface could stop the disease. The binding pattern and potency of tetra- and pentavalent CTB5 inhibitors based on the same GM1 ganglioside scaffold are compared for the first time.

X. Yao, J.-X. Ru, C. Xu, Y.-M. Liu, W. Dou, X.-L. Tang, G.-L. Zhang,* W.-S. Liu*

**Figure:**
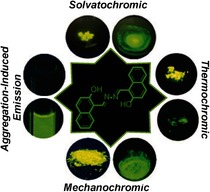


478–482

Multistimuli-Responsive Luminescence of Naphthalazine Based on Aggregation-Induced Emission

**Grind and make it hot!** A remarkable change in the fluorescence emission of 2,2-dihydroxy-1,1-naphthalazine occurs upon mechanical grinding, heating, and exposure to solvents. Its characteristic aggregation-induced emission (AIE) properties are reported herein. The fluorescence change could be a result of a transition between two structurally different polymorphs. This compound could have applications as a multistimuli- responsive luminescent material.

X. Chen, T. Mei, Y. Cui, Q. Chen, X. Liu, J. Feng, Q. Wu, D. Zhu*

**Figure:**
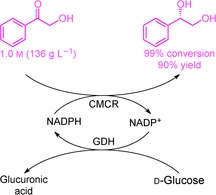


483–488

Highly Efficient Synthesis of Optically Pure (S)-1-phenyl-1,2-ethanediol by a Self-Sufficient Whole Cell Biocatalyst

**Self-sufficient catalysis!** Lyophilized recombinant Escherichia coli coexpressing Candida magnolia carbonyl reductase (CMCR) and glucose dehydrogenase (GDH) genes served as an effective selfsufficient biocatalyst for the reduction of a-hydroxy acetophenones at high substrate concentrations. The products were isolated with high yield and excellent optical purity, offering a practical biocatalytic method for the preparation of vicinal diols.

E. Mondal,* J.-P. Lellouche,* M. Naddaka

**Figure:**
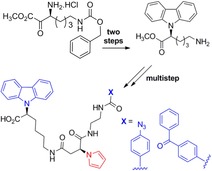


489–496

Novel Carbazole (Cbz)-Based Carboxylated Functional Monomers: Design, Synthesis, and Characterization

**Functional monomers:** Novel functional carbazole-based carboxylated monomers were accessed using a Clauson- Kaas procedure, a deprotection step, amide coupling, and hydrolysis in a multistep synthesis. The design strategy was extended to complex carbazole- COOH monomers incorporating arylazo and phenylazide or benzophenone as photoreactive moieties, which can be utilized for functionalization/decoration of various polymeric and non-polymeric surfaces, matrices, and non-functional nanomaterials in their monomeric states or as polymeric microparticles (MPs).

S. Yousuf, R. Alex, P. M. Selvakumar,* I. V. M. V. Enoch,* P. S. Subramanian, Y. Sun

**Figure:**
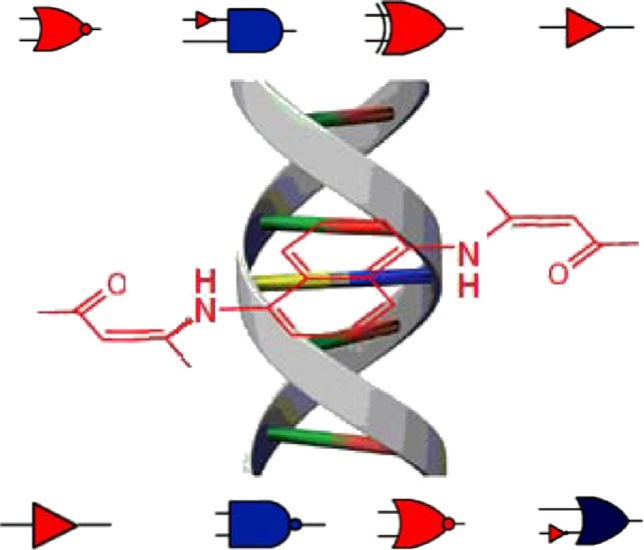


497–508

Picking Out Logic Operations in a Naphthalene b-Diketone Derivative by Using Molecular Encapsulation, Controlled Protonation, and DNA Binding

**Lifting logic from molecules:** Compounds or complexes that have different properties depending on specific inputs, e.g. pH, can be used to generate Boolean logic functions. Naphthalene bdiketone derivatives bind to DNA and b-cyclodextrin, where aminopentenone substituents are encapsulated inside the b-cyclodextrin cavity. The resulting compound displays NOR, XOR, NAND, and Buffer logic operations based on proton, cyclodextrin, and DNA as chemical inputs.

T. J. Sørensen,* L. R. Hill, S. Faulkner*

**Figure:**
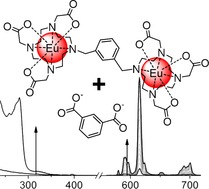


509–515

Thermodynamics of Self-Assembly of Dicarboxylate Ions with Binuclear Lanthanide Complexes

**Eu complexes assemble!** Scrutiny of the association between a binuclear lanthanide complex, 5-nitro- or 5-aminoa, a’-bis(Eu·DO3Ayl)-m-xylene (where DO3A is 1,4,7,10-tetraazacyclododecane- 1,4,7-triacetic acid), and aryl carboxylate guests shows that solvation and small perturbations to molecular structure are extremely important for the self-assembly process involving kinetically stable lanthanide complexes.

M. S. R. Bobe, M. Al Kobaisi, S. V. Bhosale,* S. V. Bhosale*

**Figure:**
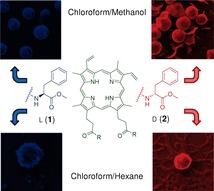


516–522

Solvent-Tuned Self-Assembled Nanostructures of Chiral l/d- Phenylalanine Derivatives of Protoporphyrin IX

**Porphyrin self-assembly:** We used land d-phenylalanine to modify protoporphyrin IX into methyl l/d-phenylalanine diamideprotoporphyrin IX (1 and 2). Solvophobic-controlled self-assembly of these chiral peptide—porphyrin derivatives led to the formation of nanostructures of various morphologies: spheres, nanofibers, lamellar structures, and thread-like and spherical shells.

M. R. Catalano, G. Cucinotta, E. Schilirý, M. Mannini,* A. Caneschi, R. Lo Nigro, E. Smecca, G. G. Condorelli, G. Malandrino*

**Figure:**
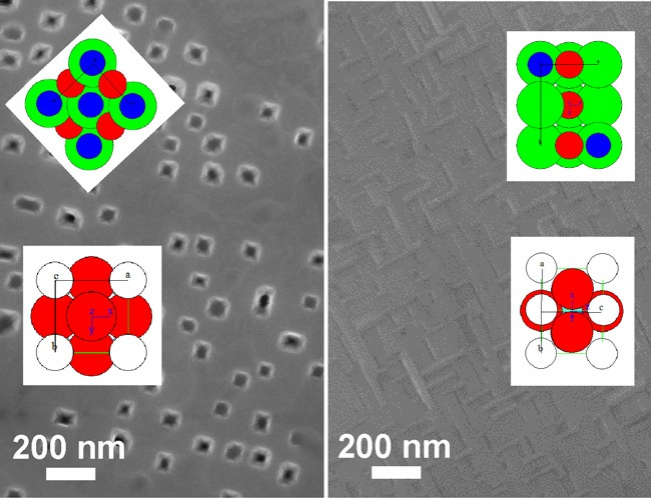


523–532

Metal-Organic Chemical Vapor Deposition (MOCVD) Synthesis of Heteroepitaxial Pr0.7Ca0.3MnO3 Films: Effects of Processing Conditions on Structural/Morphological and Functional Properties

**Heteroepitaxial growth:** Pr0.7Ca0.3MnO3 (PCMO) films were prepared on SrTiO3 (001) and SrTiO3(110) single crystal substrates though a simple metal-organic chemical vapor deposition (MOCVD) approach. SrTiO3 represents the model guide on which the perovskite structure of PCMO can replicate. The presence of a ferromagnetic transition temperature was correlated to the transport properties of the film.

## THESIS TREASURY

J. E. Stumpel*

**Figure:**
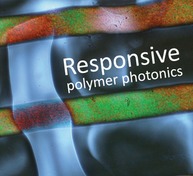


533–535

Responsive Polymer Photonics

**Stimulus-responsive materials:** This thesis describes the development of stimulus-responsive polymeric materials based on liquid crystalline polymers and hydrogels. Novel responsive molecular building blocks were designed and synthesised. Specific monomer mixtures were crosslinked by UV-photopolymerisation methods, and the response of the obtained (structured) material was analysed.

Madhuprasad*

**Figure:**
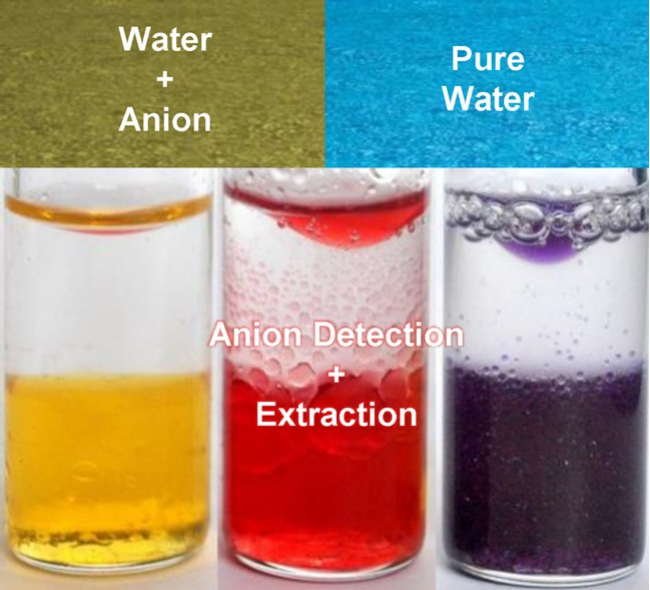


536–540

New Synthetic Receptors for Molecular Recognition of Anions and Their Practical Applications

**Heightened senses!** Anions such as fluoride play essential roles in human health; however, with increased industrialisation, many anions are becoming pollutants. As such, there is a continued need for the development of safe and efficient techniques for the detection and removal of these anions from the environment. The research summarised here focused on the synthesis of organic receptors for the detection of anions and their different application.

## SERVICE

* Author to whom correspondence should be addressed.

Supporting information is available on the WWW (see article for access details).

A video clip is available as Supporting Information on the WWW (see article for access details).

This is an open-access article, published under the terms and conditions of a Creative Commons License, as stated in the final article.

Contributions labeled with this symbol have been judged as “Very Important Papers” by the referees.

